# A David and Goliath set-up: a qualitative study of the challenges of ensuring the introduction of cost-effective new cancer medicines in Finland

**DOI:** 10.1186/s40545-022-00449-5

**Published:** 2022-08-29

**Authors:** Eeva Ollila, Vesa Kataja, Liisa Sailas

**Affiliations:** 1 Cancer Society of Finland, P.O. Box 238, 00131 Helsinki, Finland; 2 Faculty of Social Sciences, University of Tampere, Tampere, Finland; 3 Kaiku Health, Part of ELEKTA, Helsinki, Finland; 4 University of Eastern Finland, Jyväskylä, Finland; 5 Cancer Center, Joint Municipal Authority for North Karelia Social and Health Care Services (SiunSote), Joensuu, Finland

**Keywords:** Cancer medicines, Finland, Healthcare policy, Medicines’ introduction, Health technology assessment, Pricing

## Abstract

**Background:**

To combat the global challenge of cancer, priority has been placed on the research and development of new cancer medicines (NCMs). NCMs are often approved for marketing in accelerated processes. Despite significant advances in treating cancer, the overall added value and high prices of NCMs has been questioned. While market authorisations for NCMs are granted at the EU level, the assessment of added value, price negotiations and purchase or reimbursement decisions are made by member states. This article explores the practices in Finland for assessing and deciding on purchasing or reimbursing NCMs.

**Methods:**

Semi-structured interviews were conducted with 26 civil servants, hospital employees, scientists, and representatives of cancer NGOs and of the pharmaceutical industry in 2019 and 2020. The transcribed interviews were coded inductively using Atlas.ti software and analysed thematically under 3 major themes and 11 sub-themes.

**Results:**

The clinical value of NCMs is considered to be high, especially regarding NCMs for certain types of cancer. Proper patient selection is important but difficult and not all NCMs can be considered as adding value. The prices are considered to often be very high, leading to concerns about the sustainability and equity of health systems. Equity concerns among cancer patients are raised concerning differences in the availability of NCMs between hospital districts and cost differences for patients between those receiving outpatient and inpatient treatment. The systems and processes in Finland for deciding on the introduction of NCMs are fragmentary, involving separate approaches for outpatient care and hospital medicines by under-resourced evaluation bodies. The scientific evidence available is often limited for evidence-based decisions on introduction. Individual hospital districts sometimes introduce NCMs without assessment by national bodies. This can hamper the proper assessment of some NCMs before their uptake and lead to unequal access to NCMs by hospitals. There is an increasing lack of transparency about pricing, due to the rapid increase of market entry agreements. Lack of transparency on information on prices poses a challenge for authorities responsible for equitable access to cost-effective care within the available resources.

**Conclusions:**

Robust reform of the national introductory systems is needed. Internationally, efforts are needed to increase price transparency, to revise incentives within the system of market approval and to accumulate and assess evidence of comparable value and cost-effectiveness after the market approval of NCMs.

**Supplementary Information:**

The online version contains supplementary material available at 10.1186/s40545-022-00449-5.

## Background

Cancer is one of the greatest global health challenges [1] and there is increasing emphasis on the need for research and development efforts to discover improved treatments. Under the European Union (EU) Pharmaceutical Strategy and the Cancer Plan, the EU is committed to ensure that patients across the EU can access high-quality treatment and new therapies when needed [2].

For medicines to be allowed to enter the market, they must first receive market authorisation. In the EU, market authorisation can be granted through four different ways, but for new cancer medicines (NCMs) applications are to be submitted to the European Medicines Agency (EMA) (Regulation (EC) No 726/2004) [3]. The EMA assesses the safety and efficacy of new medicines but not their added value compared to other available medicines nor their cost-effectiveness.

To foster the availability of new medicines with anticipated major public health needs, incentives such as accelerated approval processes, alleviated evidence requirements, and the scope for strengthened patent protection through orphan medicine designation (Regulation EC No 141/2000) have been established [4]. NCMs are often assessed by the EMA’s accelerated assessment procedure. Some NCMs have orphan drug designations [5].

There have been significant advances in the medical treatment of certain cancers, such as melanoma [6–8] and small cell lung cancer [9–11]. Nevertheless, the overall added value of NCMs has been questioned [4, 12–16]. Furthermore, Davis et al. [4] found that 57% (39/69) of the EMA-approved NCMs entered the market without evidence of improved survival or quality of life, and after a minimum of 3.3 years of follow-up there was still a lack of conclusive evidence on those aspects. Serious concerns have been raised internationally over the high cost of NCMs, impact on cost-effectiveness, equality of cancer care, and the impact on the overall sustainability of health care systems [14, 15, 17].

Questions have been raised about the adequacy of the existing assessment, procurement, and cost management policies and practices in countries to ensure sustainable and equitable access to cost-effective cancer medicines [15, 16]. The EU has sought to strengthen current processes, including by the European Commission Regulation on health technology assessments (HTA) (EU 2021/2282). Various cross-country collaborative activities to improve access are also being discussed [18].

The financing and organising of health care are the national competence of EU member states, and it is up to them to decide on the use of public money for medicines. The added value, cost-effectiveness, and estimated budget implications of medicines and the availability of resources are estimated by the designated bodies of EU member states, and so prices are negotiated and reimbursement and purchasing decisions are made by member states. Strengthened health technology assessment mechanisms coupled with more sophisticated pricing and reimbursement systems in EU member states has been called for [19]. In Finland, the pharmaceutical industry, cancer NGOs and some cancer researchers and institutions have campaigned for the faster introduction of NCMs as well as for more personalised cancer care.

This study explores the views of Finnish experts and stakeholders on the value of NMCs and on introductory structures and processes of NCMs and on the impact of NCMs on Finnish cancer care. This study may be of wider interest, as the challenges in ensuring swift and equitable access to cost-effective cancer medicines in the context of limited hearth care and in ensuring pricing transparency are encountered and debated internationally.

## Materials and methods

This study is based on thematic interviews with 26 experts and stakeholders representing major institutions concerned with introducing medicines, scientists, non-governmental cancer-linked organisations (cancer NGOs), and the pharmaceutical industry.

### Identifying and contacting the persons to be interviewed

First, we identified the various bodies involved in introducing NCMs and their mandates, and stakeholder organisations, such as cancer NGOs and pharmaceutical companies or associations. We then identified the people for interview from each organisation based on their formal job descriptions, meaning their work in the process of introducing medicines or their role in a cancer NGO or in a pharmaceutical company or association. Furthermore, scientists with relevant work on pharmaceutical policies were identified. Some of the interviewees, including several employed by hospitals and universities, were identified based in addition to their formal work duties on their publications and presentations linked to introducing NCMs.

Potential interviewees were first contacted by email with a description of the study and invitation for interview. A written informed consent form was sent prior to the scheduled interview, signed at the start of the interview or acknowledged by email when the interview was done by phone. The characteristics of the 26 persons interviewed for this study are described in Table 1.

**Table 1:** The 26 interviewees of the study

Category	Number of interviewees
Civil servant -Employed by the Ministry of Social Affairs and Health or organisations with responsibilities concerning pharmaceuticals under its guidance, or the Social Insurance Institution of Finland (KELA)	9
Hospital employee -Employed by a university hospital, either in cancer care, the pharmacy or administration	5
Scientist -Employed by a university or a scientific journal	4
Pharmaceutical industry representative -Employed by the pharmaceutical industry or industry association	3
Cancer NGO representative -Employed by a cancer NGO	4
Total number of interviewees	26

The 26 interviewees were categorised by their main current position, although many had either prior or current positions in several professional categories. For instance, several hold concurrent positions at a university and university hospital. Civil servants, hospital employees and scientists are together referred to as experts, and cancer NGO and pharmaceutical industry representatives are together referred to as stakeholders.

Four people requested for interview declined and two did not reply to the request. Of those who declined, one NGO representative did not want to take part on the grounds that there are no problems with access to NCMs after the Managed Care Agreements (MEAs) became possible; an interview with two cancer NGO representatives was on the request of the interviewees redone with just one of the original interviewees participating; and two civil servants representing the business aspects of pharmaceutical policies could not find a suitable time for the interview.

### Conducting the interviews

The semi-structured interviews were assisted by a list of thematic questions (see Additional file 1 for the thematic questions), slightly customised for each group of interviewees and for the job description and expertise of the particular interviewee, and with some additional questions based on information from previous interviews. The interviews explored the views of the interviewees (1) on the value of NCMs in modern cancer care in terms of their clinical significance, costs and health care implications, and (2) on the processes of introducing NCMs, defined for the purposes of this work as the structures, processes and actors of introducing NCMs in outpatient and in hospital care up to the point of a decision to purchase or reimburse. In addition, views on future cancer care and the research and development (R&D) environment were discussed, the results on these discussions being reported separately.

The interviews were conducted face to face, except in three cases, which were done by phone, and all were conducted by the same person (EO). Each interview lasted about an hour (on average 63 min). Interviews were held between March and October 2019, except in one case, when the interview was done over two sessions (in December 2019 and February 2020), and in another when a second short interview was conducted with one interviewee, in October 2020, to clarify issues arising from the first interview.

### Analysing the interviews

The interviews were recorded and transcribed, except in one instance when notes were used. Interviews were coded inductively by EO using Atlas.ti software, and the codes were grouped in 11 sub-themes and 3 themes (Table 2) for this study (see also Chapman et al. [20] for grounded theory and thematic analysis). The interview data were triangulated using scientific papers, pharmaceutical policy papers and, for additional information, the websites of the relevant organisations and articles in lay magazines, some of which were identified by the interviewees themselves. The citations of the particular interviewee together with the draft preceding paragraph of each citation were sent to the interviewees, and some additional amendments were made as a result of responses received.

**Table 2:** Themes, sub-themes and codes

Themes	Sub-themes	Codes
The value of NCMs	NCM clinical significance	NMC significance NCM advances NCM challenges NCM adverse effects NCMs for hope
	NCM costs and price	NCM prices—facts NCM prices—opinions Acceptable prices for QALY Drug budgets
	NCMs and health care	Cancer care systematic issues Cancer care advances Health care resources NCM equity NCMs and cancer care dilemmas
Introduction of NCMs	EMA market approval of NCMs	EMA market approval, EMA conditional market approval
	National introductory system (as a whole)	Regulation, evaluation and introduction (general) 2-channel system Giving OC medicines from the hospital Special permission
	Introduction for outpatient care	OC evaluation and reimbursement
	Introduction for hospital care	Assessment of hospital medicines Procurement in hospitals Compassionate use
	International cooperation	International cooperation HTA Directive HTA Directive concerns
Transparency and conflicts of interest management	Transparency	Transparency
	External influences	Lobbying Media role Pharmaceutical industry influence Social pressure
	Conflicts of interest management	Conflict of interest

## Results

### Views on the value of NCMs

#### The clinical significance of NCMs

The experts interviewed agreed that cancer treatment has improved significantly over the years. Treatments were now available for many situations where previously there were none, such as for progressed cancers. Treatments were also more specific in terms of targeting cancer cells more specifically. Major advances have been made in treating many cancer types, especially melanoma, lymphoma, ovarian cancer, and certain breast cancers and lung cancers.

“For some diseases that in the past were regarded as completely desperate cases for individual patients there has been even very significant prolongations of survival.” (Hospital employee)

Major advances made in immuno-oncology and the increased availability of oral treatments was mentioned by many of the interviewees. While oral treatments enabled outpatient care, it was mentioned that some NCMs could also require longer follow-up in hospitals due to side-effects.

Often, NCMs have provided improved treatments for very specific and small patient groups. The new treatments available required new skills. They also called for new infrastructure, including very specific diagnostics for finding those patients that would benefit of any particular NCM, as even if only a portion benefitted all were exposed to potential side-effects.

There was a common view that not all NCMs added value. Some maintained that while the promises were huge they are currently often not fulfilled. Some NGO representatives mentioned that bringing hope for a critically ill cancer patient could be a reason to give an NCM, even if with low expectations for effectiveness.

#### Costs of NCMs

Many experts interviewed considered the current prices of many NCMs to be too high, even opportunistic for the society, especially considering the scarcity of data on their effectiveness. They agreed that prices, cost-effectiveness, and opportunity costs need to be discussed, even if difficult. Cancer NGOs emphasised that a strong patient perspective was needed in such discussion.

“I think that the prices by which the NCMs come on the market are very high. So how long can society really, even this kind of rich society, especially when cancer is getting more common and lifespan is getting longer, bear these costs and respond to the needs.” (Civil servant)

Costs can be considered from different perspectives: the costs of all medicines per person, costs per medicine per person, the budget implications of introducing an NCM and costs in relation to a medicine’s effect, often calculated as costs for a quality-adjusted life year (QALY). QALYs, costs and budget implications are in principle considered by the national assessment bodies Pharmaceutical Pricing Board (PPB), The Finnish Medicines Agency (Fimea) and Council for Choices in Health Care in Finland (Cohere), while hospitals seemed more focussed on costs per patient and budget implications. In all assessments costs are usually considered for single medicines, not for treatments.

“What makes it a little absurd is that often these patients have—it’s not just one medicine—but they often have five, six other medicines, which are also remarkably expensive.” (Hospital employee)

The interviewees were asked for their opinion about an acceptable cost for a QALY. All stressed that there was no official upper limit for the price of a QALY, although some unofficial upper limit guideline seemingly exists. The most cited cost was 30–40,000 €, two interviewees mentioned a somewhat lower price range, and several cited 50,000 €, and one hospital employee mentioned 100,000 €.

The experts interviewed stressed that a considerably higher price for a QALY could be accepted if the target population were small or if no other treatment existed, as is often the case regarding NCMs. But it was also pointed out that the indications for NCMs tend to become broader with time, which from the financial point of view can be problematic, but being, according to one expert, part of a deliberate strategy of the pharmaceutical industry. One expert stressed that a higher cost of an NCM were acceptable for one-time treatments with high expectations for significantly increased life expectancy or even cure, such as with the Car-T treatment. Conversely, another expert said that some considered a high cost acceptable for later stages of life, as the use was short-term. A further problem with NCMs relating to their value was that there were inadequate means for selecting patients most likely to benefit from them, therefore reducing cost-effectiveness markedly.

“So, if one in ten benefits greatly, three out of ten benefit mildly and the rest not at all, but all get the adverse effects, so in a way we cannot pay a full price for all ten… (Civil servant)

Annual costs of NCMs per patient clearly exceed 30–40 000 €, almost as a rule. One expert mentioned 70,000 € for an NCM extending life by about 4 months. Three cited costs of up to 500,000 € for one NCM. Regarding one university hospital district it was mentioned that of 20 medicines with the highest budget implications 18 were NCMs, comprising about 35% of the overall budget for hospital medicines. As NCMs typically exceed prices found acceptable for other medicines, a possibility of creating a special fund for paying for NCMs was raised by two experts.

#### Views on cancer care—sustainability and equity

The overall sustainability of health care and equity between various types of patients were particular concerns especially considering the increasing needs of the ageing population and serious gaps in many areas of health care. While pharmaceuticals do not currently form a major part of the spending in specialised care the dramatic increases in the prices of NCMs, raised serious concerns. Prioritisation in health care was acknowledged to be both necessary and difficult.

“Health care resources cannot increase forever… In some cases it can be very valuable to increase life expectance by a month. In other instances, it may be just an end time, full of side-effects and stupor, and the price tag may be 100,000 €. If you then think what else you could have done with the money in primary health care, for example 1500 visits to a doctor or something else, so you may think these kinds of question, if you think about the opportunity costs.” (Civil servant)

A need to improve collaboration between primary and specialised health care to ensure early diagnosis, integrated treatment as well as timely initiation of palliative care was recognised. It was even said that sometimes a decision to use of NCMs even with a patient that should rather be given palliative care could be influenced by poor integration between curative and palliative care making the change difficult both for the patient and the doctor.

**Fig. 1:**
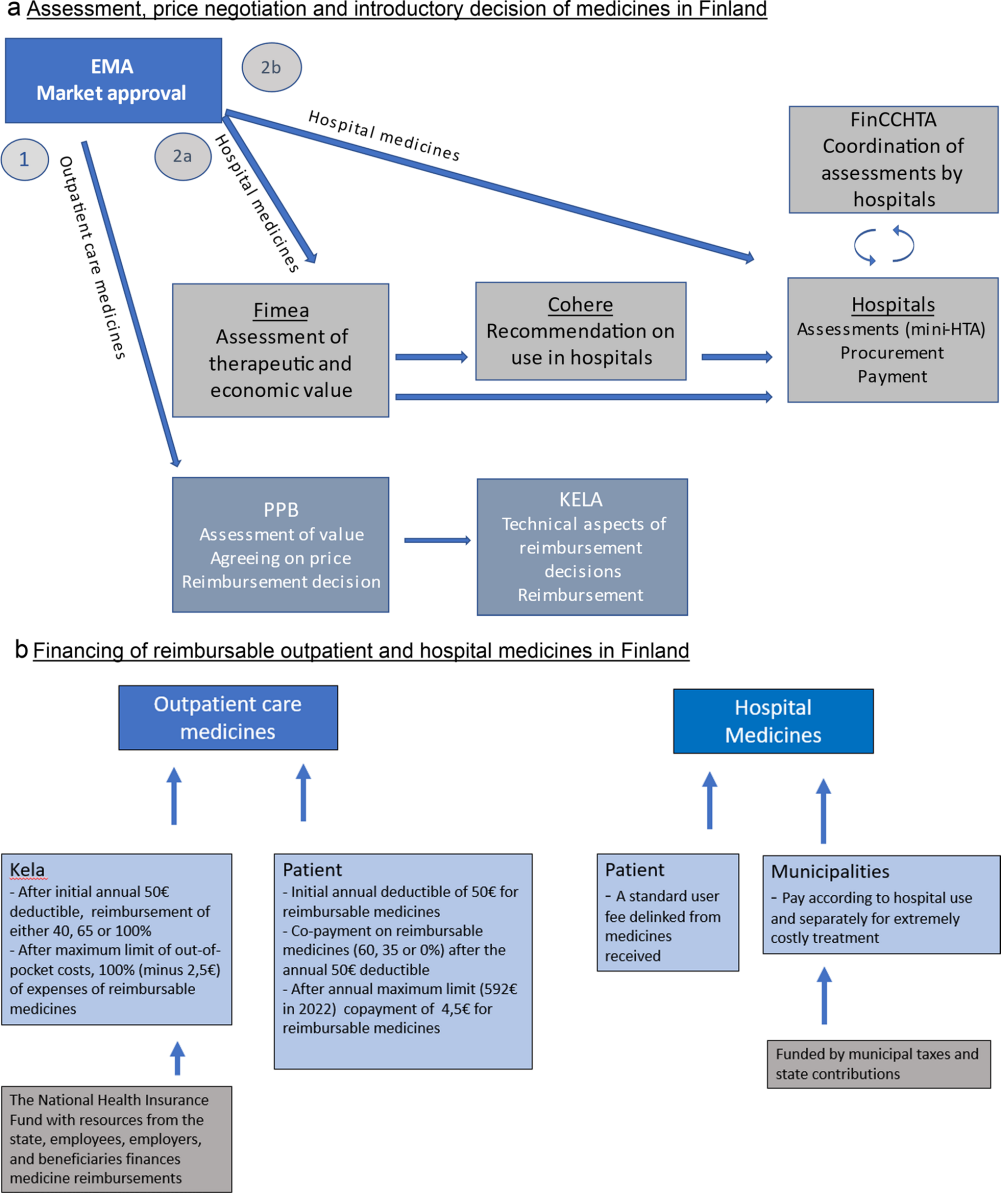
The structures for introducing and paying for medicines in Finland. **a** Assessment, price negotiation and introductory decision of medicines in Finland. **b** Financing of reimbursable outpatient and hospital medicines in Finland. After the market approval by the EMA, the assessment and reimbursement decisions for outpatient care (OC) medicines are made at national level by the Pharmaceuticals Pricing Board (PPB) and implemented by the Social Insurance Institution of Finland (KELA) (**a** Route 1). OC medicines carrying reimbursement decisions are co-financed by national funds of KELA and patients themselves (**b**). Regarding hospital medicines, the Finnish Medicines Agency (Fimea) assesses their therapeutic and economic value. The Council for Choices in Health Care (Cohere) issues national service recommendations, including hospital medicines, on what should or should not be included in different public health services (**a** Route 2a). Alternatively, if no Fimea assessment exists, the hospital districts make their own assessment, called mini-health technology assessments (mini-HTAs) (**a** route 2b). The Finnish Coordinating Center for Health Technology Assessment (FinCCHTA) coordinates the assessments done by hospitals districts. The 21 hospital districts, further grouped in to five university hospitals and financed by the municipalities, are in charge of procurement and payment of hospital medicines. During hospitalisation and with polyclinic treatment at a hospital, medicines are included in the patient fee (**b**). From the patient’s point of view, there is a marked difference in costs depending on whether the medicines are provided by the hospital or bought from the pharmacy, and the cost reimbursement is in practice a necessary prerequisite for affordable purchasing of NCMs. The various structures involved have distinct accountabilities, involving the Ministry of Social Affairs and Health (PPB, Fimea, Cohere), the Parliament (KELA) and the municipalities (hospital districts). The ongoing reform of social and health services will bring marked changes to the structure, financing and lines of accountability, with a new administrative layer, wellbeing services counties, being responsible for organising social and health services with (at least initially) national budget funding

For the patients, the costs of NCMs depend upon whether the treatment is administered in the hospital or whether it is part of an outpatient care, and if part of outpatient care, on the reimbursement status of the medicines (Fig. 1b). These differences in costs of NCMs for the patients resulting from the current two-channel system and various percentages of reimbursements were seen by many interviewees to cause major inequity among cancer patients.

Several interviewees raised the issue of inequity in current cancer care caused especially by differences in the availability of various NCMs by hospitals leading to significant geographical inequity in cancer care.

“I think it's a problem in principle that the hospital districts themselves can choose the medicines they introduce. I think it's in principle a very big problem of inequality, a very big problem.” (Cancer NGO representative)

### Introduction of NCMs

#### Views on EMA market approvals of NCMs

NCMs are often approved for market with the EMA’s accelerated assessment processes, some with Orphan drug designations. They are typically meant for very small patient groups and their approval is based on limited evidence.

“EMA marketing authorisations come with less and less evidence as medicines are used in smaller and smaller numbers of patients. A marketing authorisation application may be based on, say, only 20 or 50 patients, and studies are also increasingly 1-armed.” (Civil servant)

Granting market approvals at an earlier stage of research and development, may also actually shift attention on swift introduction of NCMs from the EMA towards national and subnational actors.

“In the past, the EMA was criticised for taking so long to process marketing authorisations. That the market access is the problem. And now the EMA has in a way accelerated it and brought it forward. And then, of course, the pricing authorities are being bombarded in a certain way because we are so rigid and nasty and slow and bureaucratic. And this sort of thing is a bit of a roundup.” (Civil servant)

One way to strengthen the evidence is by granting market approval as a condition for additional evidence within a required timeframe. However, it was also said that the withdrawal of a medicine after even conditional market approval was difficult in practice—if even a patient had benefitted from it—although it did sometimes happen. Nevertheless, the need to have additional good quality clinical was stressed, including research comparing various treatments, improving means of selecting patients that would benefit most from NCMs, as well as a general need for strengthening post-launch evidence production especially at the EU level.

The civil servants and hospital employees involved in decisions on purchasing or reimbursements of NCMs are often faced with a dilemma: on the one hand, a swift introduction of NCMs could potentially provide important benefits for certain patients; on the other, significant uncertainties exist due to the limited evidence on effects and on adverse effects, as well as insufficient means to select those patients most likely to benefit. Some interviewees questioned the suitability of the traditional health technology assessment processes for such medicines that are targeted for small patient groups.

“These kinds of innovative drugs that are for such a small group of patients, that have just one mutation. When the cancer patient groups are split into such small groups and for the effective drugs you never get that kind of clinical evidence until it’s introduced.” (Cancer NGO representative)

#### The national structures and procedures for introducing NCMs

##### The introductory system as a whole

In Finland, there are separate procedures and systems for introducing and funding medicines for outpatient care (OC) and for use in hospitals with respect to assessing, agreeing on price, and deciding on reimbursement (OC) or on purchasing (hospitals) (Fig. 1). There was a common understanding among the interviewees that the current systems for introducing medicines in Finland are fragmented and the processes are complicated, being sub-optimal for enhancing the rational use of medicines.

“At the moment, this is so fragmented that no one really understands what is happening.” (Scientist)

The two-channel system was considered to be a major problem. It was pointed out by several experts that the criteria for introducing NCMs differed between these two systems.

As some modern cancer treatments combine intravenous and oral treatment, the two-channel decision-making structure with distinctive processes and criteria was seen as especially problematic for cancer treatments in cases in which the oral treatment was not reimbursed. Based on the interviews, the problem seems often to be linked to oral NCMs, which were perhaps used in clinical trials but lacked a reimbursement decision, and especially more recently to a desired off-label use of oral NCMs. Consequently, some hospitals release oral NCMs without a reimbursement decision from the hospital pharmacy for outpatients. The practice has been discouraged by hospital chief medical officers, at least without an assessment coordinated by the Finnish Coordinating Center for Health Technology Assessment (FinCCHTA) [21], as it is not in line with the defined functions of hospital pharmacies and is in practice breaking the law. According to the interviews with experts working in hospitals, some but not all hospitals had discontinued this practice.

There was discussion on whether the criteria for market approval and subsequently for introductory decision in countries should be based on the location of a cancer or on a type of mutation. Some noted that the existence of a specific mutation did not mean that a certain pharmaceutical would be effective for all types of tumours with that mutation and called for caution in patient selection to ensure cost-effectiveness and cost-containment. Some also maintained that there should be more flexibility for off-label use in outpatient care. In the context of hospital care, the idea of precision medicine was also mentioned as justifying the more experimental use of NCMs.

“It’s one of these precision medicine ideas that you don’t just look at the molecules that have an indication for this disease, but in fact quite randomly, just like a molecule built for another disease might work for an individual patient.” (Hospital employee)

Fimea may grant special permission for a medicinal product that has no marketing authorisation to be released for use in individual cases for special therapeutic reasons, for instance in cases for which no other treatment is available or may not have the desired effect. If applied for, PPB can grant a reimbursement for such a medicine. Also, patients can seek to access NCMs without EMA marketing authorisation by enrolling themselves in a clinical trial, a crucial option according to several hospital employees and cancer NGOs and pharmaceutical industry representatives.

There is consensus on the need to reform the current systems so as to ensure expeditious good quality assessment and decision. However, there are differences of opinion among the interviewees concerning the extent to which the new systems should be national or subnational, and how to link the decision on introducing NCMs with financing. It was noted that having national funding for hospital medicines delinked from hospital budgets would likely increase the use of NCMs, especially in terms of criteria for use and patient selection potentially tilting the balance between medical and other forms of treatment. National guidance on treatment protocols were called for. In this context, two experts mentioned that there would also be a need to increase measures to ensure rational use of currently nationally funded OC NCMs.

“At the national level, as I see it, we should outline which treatments are effective and which are worthwhile introducing. But the process should be fast, not slow. And then I would say that the money should also come through a single window, so that the entire cooperation area has a common sum of money, which is then also defined so that these drugs are introduced in this area. And also so that the outpatient doctors, sorry, now doctors, would not have the right to prescribe whatever they want.” (Hospital employee)

##### Introduction of NCMs in OC

The process for deciding on the reimbursement of OC medicines at the PPB and reimbursements by Social Insurance Institution of Finland (KELA) was considered to be long and complicated (Fig. 1a). The use of MEAs, also called risk-sharing agreements, became possible for OC medicines in 2017, after which delays were no longer seen as the major problem by most of the subjects interviewed.

“Criticism over delays has been quite justified in the sense of how we got these new medicines to patients. So it just started to take too long.” (Civil servant)

“The situation is improving for these outpatient medicines. The most obvious reason is the practice of risk-sharing agreements.” (Pharmaceutical industry representative).

It was stressed by the PPB that the timeframe from EMA market approval to reimbursement decision involves many delays, from submitting an application to the PPB to requesting further information and accepting the suggested price, and regarding MEAs agreeing on the price. It was mentioned that Finland was not among the first countries where reimbursement applications would typically be submitted.

The fact that MEAs were in use in most other EU countries but not in Finland until 2017 exacerbated the challenge of providing a swift and cost-effective access to NCMs, as the authorities in Finland dealt with the list prices in their deliberations, while the prices actually paid in other countries were presumably lower. Since 2017, most reimbursement decisions on NCMs have been made with MEAs. According to the PPB website, NCMs have made up 58% (101/173) of all MEAs made for OC medicines by May 2022. All were economic MEAs, meaning that they included a simple undisclosed price reduction as compared to the public list price. Performance based MEAs were said to pose many practical difficulties and are not expected to be used for OC medicines. The list price of the NCM with MEA is initially paid and the company returns the difference to KELA. Secrecy surrounding the actual price paid is ensured by releasing only the pooled total sums of these return payments for all medicines with MEAs.

Although before 2017 there were no specific price negotiations by the PPB, a major issue in the reimbursement decision process concerned weighing the clinical value of the medicine and the reasonability of the suggested wholesale price. MEA prices are subject to negotiations, which are done by the regular staff as part of their routine duties. There is an enormous asymmetry of information, power and experience concerning pricing between the negotiators.

“A David and Goliath set-up. They know how they have snatched all the big countries from the European market—Germany, France, Great Britain, probably Sweden too, which would be where we are, but we don’t know what deals they have made there; we don’t know what the price level is there.” (Scientist)

##### Introducing NCMs in hospitals

The Finnish Medicines Agency (Fimea) and the Council for Choices in Health Care in Finland (Cohere) are the main channel for assessing the NCMs for hospitals (Fig. 1a). Fimea does not have the resources to assess all NCMs and Cohere’s resources for issuing recommendations are even more limited, leaving hospitals to do some of the assessments themselves. Although Fimea aims to anticipate EMA market approvals and have its assessments ready shortly after the market approval is granted, some experts indicated that the process through Fimea and Cohere was too slow, while others seemed more willing to wait for Fimea assessments and for Cohere recommendations, which the hospitals would take seriously, even if they are not binding.

When there is no Fimea assessment, hospitals can make their own assessments on a smaller scale (called mini-HTAs) with very limited expert resources. At the time of conducting the interviews, a chief medical officer for assessments was employed at four out of five university hospital districts, yet hospitals may lack any health economics expertise. The Coordinating Center for Health Technology Assessment (FinCCHTA) coordinates the assessments made in university hospital districts and seeks to streamline their structures and processes, which according to the interviews still varied and could be inaccurately defined or carried out.

In some hospital districts, the mini-HTAs were discussed before a procurement decision by a designated assessment group, but in at least one university hospital district the assessment group typically provided the information after the procurement had already taken place.

“The assessment group is not such an active organ that actually would assess these issues, it is more a body to discuss these issues post festum.” (Civil servant)

The chief medical officers of the five university hospital districts have stated that expensive medicines, defined as costing annually 30,000 € or more per person should be assessed either by Cohere or FinCCHTA, and that all hospitals should follow these recommendations [21]. According to the interviews, this may not always be the case in practice, but at least in some hospital districts the in-house decision-making process varied according to the estimated costs, with the hospital leadership being involved in making procurement decisions of the most expensive medicines.

“So we have [in our hospital] a new cancer drug treatment—if the annual cost is more than 30,000 euros per patient, its introduction must go through a specific procedures, that is, if it is now somewhere already in Cohere or Fimea processing and whether it is already in use somewhere else in the University hospitals, so it must be thought that no individual doctor may make a decision to take an expensive new drug before it is so granted. It has [evolved] perhaps now in this last 5 years.” (Hospital employee)

In at least some hospital districts, the need to assess NCMs typically arose from a potential procurement need for an individual patient. In such cases, the official processes may not be fully followed.

Q: “I wonder how often you have a situation where you have an individual patient, a cancer patient is a pretty good example, who has an acute crisis, and you go out to get the patient a drug first and that’s how the contract is created.”

A: “That is perhaps unfortunately a fairly typical situation.”

Q: “And then you don’t necessarily wait for Fimea’s and Cohere’s position?”

A: “Not necessarily, no. So and here it in a way the problem is, when the system is this kind of fragmented or has fragmented practices, so then the situation can just be formed, so that it may be even a pharmaceutical company representative, who then approaches that you have this kind of patient and he needs this and the doctor also wants to use it …” (Civil servant)

Compassionate use, meaning treatment using NCMs before EMA market approval, is also practised at least by some hospitals. This form of introduction may be prompted by a clinician, but the pharmaceutical industry may also play an active part.

Q: “If we still go through that once again so that I understand how in your hospital district, for example, these new cancer drugs or any kind of drugs are procured.”

A: “It has been needs-based in practice so far, i.e. the first patient that comes to us for whom the clinician determines the need, that this patient could be suitable for this kind of drug treatment, for which a new product has now emerged somewhere in the world and it is either already approved for marketing, but quite often today it is something that has only just received FDA recommendation: and the EMA is still evaluating it. So we would need it perhaps as compassionate use and companies know how to use this situation very well, that is before the market launch, the pharmaceutical companies are contacted and get the drug in practice for free to try it out and start on a patient.” (Hospital employee)

It was stressed that initiating the use of an NCM for an individual patient or some patients in one hospital—possibly as part of early access programmes or special offers (see also [21])—had wider implications as other hospitals would feel pressured to procure and provide the same treatment for their similar patients for reasons of equity even if they did not consider doing so otherwise justified.

“That’s the weak point of the system, because the industry is certainly taking advantage of it by pushing these so-called good deals through the side door. And that, of course, really increases inequality.” (Scientist)

Several of the oncological experts interviewed who are employed by hospitals complained that early introduction was often done in the smaller hospitals, but as all those interviewed work in the university hospital settings, we did not hear the perspectives of experts at smaller hospitals. The experts interviewed hoped that national-level assessments and recommendations will provide support for the hospitals as well as individual clinicians in their difficult deliberations, often concerning individual patients.

“When I look at our consumption statistics, I know that there are a lot of cancer medicines that are not effective and should not be used, and so in a way, they take resources away from accommodating new expensive treatments. So all the other university hospital districts figures show zero, but then there are three central hospitals where there are sales and it’s not a coincidence. …It’s also not in the interest of those patients, in a way, that people are exposed to ineffective treatments. And I don’t see it as a good phenomenon at all, I see it as a real problem.” (Scientist)

Regarding individual patients that have received their NCMs free of charge through exceptional routes, such as compassionate use or as a participant in a clinical trial, some interviewees stressed that they should be guaranteed their medications for free as long as they need them without expectations that the medicines would be paid for by the public sector. This is not always the case.

“But that’s where we start a medicine for free and the agreement is practically without exception that when it gets marketing authorisation and a price is set for it, then it becomes chargeable, so we can’t stop the medicine, if the patient dies if we stop the drug, which creates an ethical problem because it comes with a price.” (Hospital employee)

Currently, the bulk of hospital medicines are purchased via public procurement procedures that encompass most required medicines and are undertaken by university hospital districts every few years, typically including options for additional years. The procurement processes include in principle all pharmaceuticals used by hospitals, even NCMs which are not subject to competition. Some selection between NCMs with similar indications and effectiveness may be made based on prices. Also, hospital districts may organise procurement processes for some NCM categories more frequently than with other pharmaceuticals.

The personnel and resources for the procurement process are scarce. While price competition is a core idea of the public procurement process, there are no separate price negotiations within that process.

“We don’t have a system in Finland where the prices of medicines are negotiated. We have procurement, which is based on the fact that the seller offers something and we then practically accept it. And it is so out of our hands. …If we do not reach an agreement, then the agreement will not be reached. In principle, no one negotiates about hospital drugs.” (Scientist)

As mentioned, many NCMs are first procured outside the public procurement processes, often based on the needs of an individual patient. In such cases, the hospital pharmacy is charged with contacting the pharmaceutical company, agreeing on the price and procuring the medicine.

The experts interviewed maintained that MEAs were still relatively uncommon in hospital settings (see also [22]). However, their use has since increased rapidly [23]. According to the interviewees, the real price of the NCMs with MEAs was said to remain known to only a very few people. While economic MEAs that involve only a price reduction were common, some aspects concerning effectiveness could be included (see also (23) on types of current MEAs). Some experts foresaw the possibilities of further price reductions linked to releasing data collected from the normal clinical practice, also referred to as real world data, in exchange (see also [25]).

Many of the experts interviewed call for a unified national system for assessment, price negotiation and procurement. In September 2020, it was announced that there would be systematic nationwide coordination among university hospitals for negotiating and procuring new and expensive medicines [24].

##### International collaboration on assessment, price negotiation and purchasing

There is a certain amount of cooperation in health technology assessment among authorities in Finland (Fimea), Norway and Sweden, known as FINOSE. This is subject to a pharmaceutical company opting to participate in it and it applies to all three countries.

The general aim of the proposal of the European Commission for a regulation concerning health technology assessment (COM (2018) 51 final—now approved with some amendments as (EU) 2021/2282) and involving assessing the relative effectiveness of medicines at the EU level—was viewed positively by the interviewees. The wider expertise available at EU level rather than relying only on national experts was welcomed. Since the data for the EU-level assessment will be essentially the same as that submitted to the EMA, there were concerns over the added value of the process, whether comparisons would be done to appropriate alternative treatments, about the means of including new emerging data in the process, and the length of an EU-level process. The main concern was the binding nature of the original proposal. Some experts highlighted the disparities in health care systems and their financing and the national competences involved. The possibility to make national assessments and national decisions was considered important by the experts, while representatives of the pharmaceutical industry favoured the more binding nature of the original proposal.

“Its main objective is to make centralised assessments of the available data, … And then we will have the European special experts just for the specific indication and the drug, so that we get the best expertise to assess the evidence on effectiveness? What are the harms and uncertainties? I think it is in everyone’s interest if this is successful. But the decision-making process, how we weigh all the evidence in each country must be independent of each country.” (Civil servant)

The experts interviewed were not optimistic about common procurement involving a larger number of countries anytime soon, at least not outside the Nordic countries. They mentioned challenges stemming from differences in the organisation and financing of health care, including levels of national affluence. Furthermore, the pharmaceutical industry, as well as large EU countries with their own industry or with more leverage on prices were said to oppose such an effort.

### Transparency and conflict-of-interest management

As a rule, Finnish legislation provides for the availability of information of official documents with exceptions such as those regarding trade and business secrets, but the practices for ensuring the availability of public information on NCM assessments and costs could be improved. The memoranda from the PPB are in principle public and available on request after the trade and business secrets have been omitted, but they are not published. Fimea and Cohere make public reports, but there are also insufficient channels for hospitals to inform each other, Fimea and Cohere about the prices they actually pay for medicines, even when that information was public.

However, the secrecy surrounding MEAs is the most severe problem of transparency hampering the possibilities for information sharing, including for prioritisation, cost-effectiveness analysis and statistics on the costs of various medicines, as the costs are not known. With the increasing use of MEAs the problem is getting more pronounced.

The authorities’ decisions can be influenced by social pressure. For example, pressure was felt by the authorities in relation to media coverage and campaigns by the pharmaceutical industry, patient NGOs, clinicians and other experts, often involving individual patient’s stories in which the patient could be helped by the new medicine being considered by the authorities. The authorities involved do not engage in public discussion about ongoing processes.

“At some point that social pressure will come. I'm not talking exclusively about cancer drugs, but there are probably cancer drugs here too, but you can see it, for example, when you follow something like the news or somewhere [in the media], when you start seeing patients saying that there is a new drug coming out in the world and then there are of course some shocking human stories in it, so you can guess that okay soon there will probably be something like this out.” (Civil servant)

As mentioned above, after one hospital had introduced a NCM—often in the context of an exceptional route—the information on the availability of a particular NCM spread rapidly and the pressure to introduce that particular NCM was experienced in the other hospitals too for equity reasons, but the context, including the costs, of introducing the particular NCM are not necessarily disclosed. Similarly, oral medicines may be introduced as part of a clinical trial or an early access programme on the understanding that continuation of the treatment will be through reimbursed NCMs. If the reimbursement decision is delayed or even denied, for example for price reasons, the situation becomes cumbersome.

“I felt it was a terribly unfair way for the industry to act, that they are starting some kind of early access programmes here, whether they are studies or whatever. And then in a certain way it creates a need for the drug when patients start to be treated with it. And then they submit an application to us. And they have, they may have a deal with the hospital that they're going to let this early access program be in effect until that drug gets reimbursed. And then, if we get a rejection decision, well then they make a new application. And then we start getting phone calls from pharmaceutical companies.” (Civil servant)

Adequate conflict-of-interest management was mentioned as requiring greater attention by several experts, although there were differences in opinion among the various actors. For a small country like Finland there is a relatively small number of experts skilled in assessing very complicated issues concerning NCMs, and many of those with specialised knowledge have acquired their knowledge of a particular NCM when working for a pharmaceutical company producing it. Many experts also have a role in several bodies involved in decision-making on NCM introduction. The secrecy surrounding MEA pricing plus pressures to expeditious introduction may pose further challenges in ensuring that public health interests remain at the core of the decision-making.

“We have often found it difficult to find clinical experts for these cancer treatment evaluations because of their affiliations… there is no other area of society where you take money, you do it both ways. You can’t be on the board of a corporate Medical Advisor and then make a procurement decision at the same time, it doesn’t look very good if that's how you do it.” (Civil servant)

## Discussion

This study has found that the Finnish experts and stakeholders perceive the clinical value of NCMs high, especially as concerns NCMs for certain types of cancer, but many also maintain that not all NCMs add value. The scientific evidence available at the time of deciding on the introduction has often been limited to make the distinction between NCMs that add value and those that did not. The prices are considered to often be very high, leading to concerns about the sustainability and equity of health systems.

The study also found that in Finland the structures and processes for making decisions on introducing new medicines and negotiating their prices are fragmented and poorly resourced. Together with insufficient transparency, the systems leave room for inappropriate influencing, including by pharmaceutical companies.

The reforms needed in Finland include rectifying the two-channel system that separates the processes between OC and hospital medicines and splits the tasks among several entities and ensuring adequate resources for swift national-level assessments, price negotiations and procurements. This should enable timely introduction of cost-effective NCMs across the country while rejecting the introduction of those without evidence of cost-effectiveness and added value. The need for mini-HTAs in individual hospitals should be eliminated.

The options for correcting the two-channel system and for ensuring the best use of medicines with appropriate patient selection both in hospital and outpatient care are being reviewed [24, 25]. More recently, hospitals districts have embarked on a national process for medicine’s procurement concerning expensive medicines and price negotiation based on Fimea assessments and Cohere recommendations. Nevertheless, the need to implement major changes still remains.

This study shows that the authorities may feel inappropriately pressured by campaigns advocating for access to certain medicines as the authorities do not feel that they can engage in discussions involving the value and the requested price of any medicine under their ongoing assessment. As intended such pressure can affect the decision of the authorities, but that influence may not always be in the public health interest. Increased transparency on the processes leading to introduction of medicines, as well as stringent management of conflicts of interests in those processes are needed.

International collaboration in assessing new medicines and purchasing expensive medicines has been recommended, and steps in that direction have been taken [18, 26, 27]. This study detected an overall positive attitude towards future EU-level assessment on the lines of the approved HTA regulation (EU 2021/2282). As the linkages between oncologists and pharmaceutical industry are common [28], expanding the pool of independent experts through international cooperation was valued, while it was noted that there remain problems due to limited evidence.

The urgent need for more research on NCMs, especially after market approval, including comparing the cost-effectiveness of various treatments raised by the experts of this study has been discussed internationally [4, 29, 30]. Patients in clinical trials typically differ from average patients in regular practice. For example, it has been found that patients with advanced solid cancers and poor performance status may not benefit from the advanced immunotherapies [31]. Improved means of identifying those patients that are most likely to benefit from the NCMs are needed.

Some interviewees maintain that experimental use of NCMs should be possible in regular hospital care. However, when used in the regular clinical settings, and not in research setting systematic evidence is not increasing. The question remains as to how far public health services should pay for treatment that is experimental rather than being free of charge in the context of compassionate use or financed from research linked budgets with systematic evidence production.

High prices of NCMs have raised concerns about the sustainability of the overall health service system internationally [14, 16, 17]. Despite new and more expensive medicines entering clinical use and increasing costs of medicines already in the early 2000s, the overall costs of cancer care per patient had not risen in Finland between 2004 and 2014, due to the increased possibilities for outpatient care [32], but the prices of cancer medicines have increased sharply in recent years [33–35].

In this study, inequities were identified between patients suffering of various diseases, between cancer patients attending to various hospital districts regarding access to various NCMs and between patients receiving their NCMs in inpatient or outpatient care regarding their costs. Internationally, differences on the availability of NCMs across countries are often seen as a concern of inequity [2, 16]. While it is true that the pharmaceutical industry does not initially put their NCMs on the market in all EU countries, differences in the availability of NCMs could also result from assessing the added value of the NCM in the context of limited health care resources and the requested price.

Measures to deal with the high prices of NCMs have included special funds and MEAs [19]. The use of MEAs is rapidly increasing in Finland for NCMs, both in outpatient care and in hospital care, and has brought about speedier access to NCMs at least in OC. As is the case internationally [36], the MEAs made in Finland concerned price reductions rather than shared risks the usual price reductions being of similar magnitude (20–40%) reported internationally [37]. However, according to Gamba et al. [38], MEAs have led to higher list prices, a possibility also raised by the experts interviewed for this study, therefore decreasing the relevance of the reduction percentage from the list price.

The leverage of entities to negotiate prices varies [26, 37]. Larger entities for purchasing have been called for [27]. While early introducers may receive especially good deals, these may provide leverage for the pharmaceutical industry in its negotiations with other actors, as also shown in this study. In Finland, a partial solution for this is being sought by advancing the national procurement of expensive hospital medicines [24].

There have been calls for increased transparency about price setting and prices [14–16, 27, 39], and the decreasing transparency concerning the greater use of MEAs is seen as detrimental to the health systems worldwide [37]. The widespread use of MEAs hampers the accumulation of evidence, especially on cost-effectiveness, hinders prioritisation efforts for health care treatments and makes good quality statistics on pharmaceutical spending impossible. The use of MEAs places also new demands on expert, administrative and reporting capacities [23]. There is the scope and need internationally to improve the quality of MEAs from the public interest point of view [27, 36, 37].

A comprehensive review of the current incentive structure for the research and development for new medicines [40] as well as means to ensure increased transparency is necessary. Ideally, there should be sufficient information on the value of new medicines so as to enable evidence-informed decisions and prices paid for medicines by the public money should be public information.

### Limitations of the study

This qualitative study aims to draw a broad picture of the major issues in introducing new cancer medicines in Finland. A major purpose for describing the perceived value of NCMs was to provide a basis of reflection for the evaluating the functioning of the introductory systems rather than to describe the value of any particular of NCM. As a qualitative study it is not able to make justice for all types of NCMs. Neither does it quantify the opportunities and challenges encountered in the introductory systems. Nevertheless, this study provides a general view of the issues to explore while reforming the introductory systems in Finland and at the EU level.

## Conclusions

Increasing cancer incidence and prevailing deficiencies in effective treatments underscore the need for improved cancer treatments. The increasing number of NCMs given market approval with limited evidence available, high price requests and lack of transparency concerning pricing and prices pose a difficult challenge to those bodies responsible not only for their assessment, price negotiation and introductory decisions, but also for ensuring the best possible care for all within the available resources for health care [15, 16, 29].

Robust reform of the national introductory systems in Finland is needed. Furthermore, a review of the incentive structure for the research and development for NCMs in the EU is necessary so as to mitigate the challenges on limited evidence and high prices [40]. Internationally, efforts are needed to increase price transparency, and to accumulate and assess evidence also after the market approval of NCMs.

## Supplementary Information

**Additional file 1.** The interview questions.

## Data Availability

The consent ensures the anonymity of the interviewees and that the interviews will be kept confidential.

## Abbreviations

Cancer NGOs: Non-governmental cancer-linked organisations
Cohere: Council for Choices in Health Care in Finland
EMA: European Medicines Agency
EU: European Union
Fimea: The Finnish Medicines Agency
FinCCHTA: Coordinating Center for Health Technology Assessment in Finland
FINOSE: HTA collaboration network between Fimea (Finland), NoMA (Norway) and TLV (Sweden)
HTA: Health technology assessments
KELA: Social Insurance Institution of Finland
MEA: Market entry agreement
NCM: New cancer medicine
OC: Outpatient care
PPB: Pharmaceutical Pricing Board
QALY: Quality-adjusted life year
