# Supplementary material for: A David and Goliath set-up: a qualitative study of the challenges of ensuring the introduction of cost-effective new cancer medicines in Finland
Source: J Pharm Policy Pract. 2022 Aug 29;15:52. doi: 10.1186/s40545-022-00449-5 (PMC9422122; doi:10.1186/s40545-022-00449-5)
Supplement: Supplementary file 1 — Additional file 1. The interview questions. [file 40545_2022_449_MOESM1_ESM.docx]

**Questions for interviewees - a generic list**

**Background information**

- Background information on the interviewee: job title, previous work experience, education
- Background and role of the organisation, in particular in relation to new cancer medicines

**Significance and cost of new cancer medicines**

- What major new cancer medicines have become available in the last 10 years? What are their main advantages compared to previous medicines? What would you like to say about new cancer medicines in general?
- How much do you think a new treatment could cost (in quality-of-life years) per patient to introduce? How is the price determined? What would you say about the price/quality ratio of new cancer medicines for society/patients?
- Depending on the interviewee, the question of the development of cancer treatments in general

**Introduction of new cancer medicines**

- What is your perception of the introduction of new cancer medicines in Finland? How does it compare with other countries?
- What is the introduction process like in outpatient care? How has it developed/should it develop?
- What is the introduction process like concerning inpatient medicines? How has it/should it evolve? Introductory in hospitals, is the introductory process consistent across hospital districts?
- What is the role of managed entry agreements?
- What is the role of clinical trials in Finland in the introduction of new medicines?
- Depending on the interviewee, more specific questions on the roles of the different bodies in the national processes, EMA marketing authorisations, HTA regulation and post-marketing surveillance of new medicines.

**Actors**

- Who are the key players in cancer medicine policy?
- Depending on the interviewee, more detailed questions on the objectives and activities of the actors, as well as on cooperation and criteria for cooperation between the different actors

**The future of cancer care, clinical trials and the innovation landscape**

- What are the most important actions to improve cancer care in the coming years?
- What will be the main innovations in the coming years? How do you expect costs to evolve?
- Depending on the interviewee, what is the Finnish innovation environment like for pharmaceuticals? How do you think it will or should develop? What factors contribute to or impede the conduct of clinical trials in Finland? How should the contribution of the Finnish healthcare system and Finnish patients to clinical trials be evident?
